# Long-term pulmonary sequelae and convalescent immune reactions in mild to moderate COVID-19 patients during the active treatment era

**DOI:** 10.1371/journal.pone.0325379

**Published:** 2025-06-05

**Authors:** Minkyeong Lee, Byoung Kwon Park, Dong Hoon Shin, Hong Sang Oh, Chae-Hong Jeong, So-Young Lee, Jungyeon Kim, Sang-Won Park

**Affiliations:** 1 Department of Internal Medicine, Seoul National University College of Medicine & Boramae Medical Center, Seoul, Republic of Korea; 2 Center for Emerging Virus Research, Korea National Institute of Health, Korea Disease Control and Prevention Agency, Cheongju, Republic of Korea; Waseda University: Waseda Daigaku, JAPAN

## Abstract

**Background:**

During the COVID-19 endemic phase, pulmonary sequelae substantially contributed to disease burden. Immunologic responses may be critical in both acute COVID-19 and in long-term sequelae. We aimed to evaluate associations between convalescent neutralizing antibodies and long-term pulmonary sequelae in patients hospitalized with mild to moderate COVID-19.

**Methods:**

Among patients who recovered from hospitalization due to COVID-19, those who consented to participate in the study provided convalescent serum between June 2021 and April 2022. These baseline patients were invited for a second follow-up visit between September and November 2023. A serum sample was collected at the second visit, and low-dose chest computed tomography (CT) was performed. Pulmonary sequelae were defined as findings of fibrotic, fibrotic-like, and ground-glass opacities (GGOs). Antibody and cytokine levels were assessed in serum samples from the baseline convalescent phase, and antibody levels were also measured in the serum sample at the second visit.

**Results:**

A total of 107 patients were enrolled at baseline, and 37 consented to the second follow-up visit. Most second-visit patients (97.3%, 36/37) did not require an oxygen supply beyond that provided via masks or nasal prongs. Twenty-two patients (59.5%) exhibited pulmonary sequelae on chest CT at a median follow-up period of 27 months (interquartile range 25–28, range 22−30) after hospitalization for COVID-19. Fifteen patients (40.5%) had fibrotic or fibrotic-like pulmonary changes, and twelve (32.4%) had GGOs. Pulmonary sequelae were associated with older age (adjusted odds ratio 1.130, 95% confidence interval 1.028–1.243; P = 0.011). There were no significant differences in convalescent cytokines or neutralizing antibodies between patients with pulmonary sequelae and those without.

**Conclusion:**

Pulmonary sequelae were quite common on chest CT after two years of mild to moderate COVID-19 and were associated with older age. The immunological or inflammatory status in the immediate post-acute infection period did not predict long-term complications.

## Introduction

Coronavirus disease 2019 (COVID-19) has entered an endemic phase that is still experiencing multiple regional peaks following the devastating global epidemic that began in 2020 [1]. Vaccines and a few specific therapeutic options have made COVID-19 more manageable; however, the disease burden is still high due to sequelae such as pulmonary complications or long COVID, also referred to as post-acute COVID-19 syndrome. It was reported that 39–82% of moderate to severe COVID-19 patients in early 2020, when vaccination and appropriate antiviral agents were not yet introduced, had radiologic abnormalities even two years after infection [2,3].

Immunologic and inflammatory reactions are known to play critical roles in the pathogenesis of COVID-19 [4]. Vaccination or natural infection induce protective immunity that helps prevent short-term infections and substantially reduces the risk of severe complications, with the level of protection varying depending on the combination of both factors [5]. Neutralizing antibodies are crucial for predicting vaccine efficacy and overall protective immunity [6]; however, the conventional virus neutralization test (VNT) is labor intensive and requires strict biosafety measures, hindering commercial use in real-world clinical practice. Recent enzyme-linked immunosorbent assay (ELISA)-based surrogate virus neutralization tests (sVNTs) may serve as viable alternatives to assess neutralizing antibodies in clinical settings, given their favorable correlation with conventional VNTs [7].

Pulmonary sequelae after COVID-19 are clinically relevant, as they are known to be associated with respiratory symptoms and functional impairment, with a diffusing capacity of the lungs for carbon monoxide <80% [2]. However, data on long-term pulmonary sequelae of COVID-19 in the period following vaccination and antiviral treatment introduction are limited, and most existing reports focus on critically ill patients. To properly manage pulmonary sequelae after COVID-19, it is necessary to accumulate data on patients with mild to moderate COVID-19, who account for a larger portion of COVID-19 survivors.

It has been suggested that COVID-19 and idiopathic pulmonary fibrosis (IPF) share genetic, molecular, and epidemiological risk factors and mechanisms of immune-mediated injury to alveolar cells [8]. In this context, several cytokines involved in pulmonary fibrosis have been confirmed to be associated with COVID-19 and pulmonary sequelae [9]. These findings suggest that there may be relationships between the immune response at the time of infection, as revealed by antibody and cytokine responses, and long-term pulmonary sequelae after COVID-19, but further research is needed.

This study aimed to investigate inflammatory and immune responses, including neutralizing antibodies, in the convalescent phase of patients hospitalized with mild to moderate COVID-19 and evaluate their associations with long-term pulmonary sequelae and long COVID. In contrast to previous reports conducted before the introduction of vaccination and specific antiviral therapeutics, the study patients were recruited during the active treatment era, receiving steroids and antiviral agents.

## Methods

### Study subjects and design

**Fig 1:**
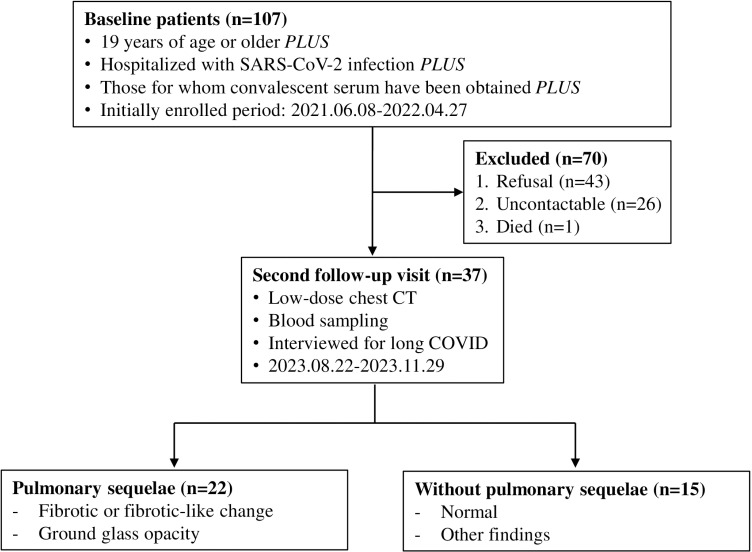
Study design.

Patients aged 19 years or older who recovered from hospitalization due to laboratory-confirmed SARS-CoV-2 infection, diagnosed by real-time reverse transcription polymerase chain reaction (rRT-PCR), were initially screened for enrollment. Patients who consented to participate in the study provided convalescent serum at enrollment between June 8^th^, 2021 and April 27^th^, 2022. These baseline patients were later invited for a 2^nd^ follow-up visit, which took place between August 22^nd^, and November 29^th^, 2023 (Fig 1).

For baseline patients, data on demographics, underlying diseases, and clinical and laboratory variables during hospitalization were retrospectively obtained from electronic medical records between January 31^st^, 2024 and February 2^nd^, 2024. The most abnormal values of the clinical data were selected. Specific therapeutic treatments and time variables related to symptom onset, diagnosis, discharge, and convalescent blood sampling were also collected. Data on vaccination status were obtained from the Integrated Vaccination Management System of the Korea Disease Control and Prevention Agency. At the 2^nd^ follow-up visit, data on pulmonary sequelae and long COVID were collected. Pulmonary sequelae data included low-dose chest computed tomography (CT) findings, resting percutaneous oxygen saturation (SpO_2_), and respiratory symptoms. Long COVID data included physical and mental recovery, changes in occupation and working hours after hospitalization, and various long COVID-related symptoms. At this visit, serum samples for the follow-up profiles of antibodies were also obtained. A history of additional SARS-CoV-2 infection during the follow-up period between baseline and the 2^nd^ visit was also recorded.

The primary outcome variable was the presence of long-term pulmonary sequelae on the chest CT scan. On the 2^nd^ visit, patients were divided into two groups according to the presence of pulmonary sequelae. Factors associated with long-term pulmonary sequelae, including cytokine levels and antibody status, were investigated.

### Definitions

Pulmonary sequelae following SARS-CoV-2 infection were defined as fibrotic, fibrotic-like, and ground-glass opacity (GGO) findings on low-dose chest CT scans [10]. The fibrotic findings consisted of traction bronchiectasis and honeycombing, while fibrotic-like findings included parenchymal bands, bronchiectasis, and linear atelectasis [10]. Three independent radiologists, who were not involved in this study, reviewed the chest CT scans along with the provided clinical information on the patient’s history of SARS-CoV-2 infection. Fibrotic or fibrotic-like changes were localized to small portions of the lung compared to GGOs, limiting the clinical relevance of describing unilateral or bilateral involvement in these findings. Therefore, in the chest CT scan results, we described unilateral or bilateral involvement only in GGOs.

Data on the history of SARS-CoV-2 reinfection were collected based on self-reported diagnoses confirmed by rRT-PCR or rapid antigen tests and met the criteria for SARS-CoV-2 reinfection by the U.S. Centers for Disease Control and Prevention [11]. The severity of COVID-19 during hospitalization was defined using the World Health Organization (WHO) severity scale [12]. The psychiatric disorders included mood disorders, anxiety disorders, and panic disorders. Fully vaccinated status was defined as two or more weeks after receiving the second dose of a two-dose SARS-CoV-2 vaccine series or one dose of a single-dose SARS-CoV-2 vaccine.

Long COVID was defined as a condition in which various symptoms usually developed three months after SARS-CoV-2 infection, persisting for at least two months, and cannot be explained by an alternative diagnosis [13]. We investigated the spectrum of symptoms listed in S1 Table. For symptomatic patients, the severity was quantified using appropriate tools for each symptom: fatigue using the Functional Assessment of Chronic Illness Therapy-Fatigue scale, dyspnea using the modified Medical Research Council (mMRC) grade and Dyspnea 12 scale, and anxiety using the Generalized Anxiety Disorder-7 [14–17].

### Measuring surface and nuclear antibodies and neutralizing antibodies

The serum sample was centrifuged at 3000 rpm for 10 minutes. The supernatant was aliquoted and stored at −80 °C. The levels of binding antibodies, IgG antibodies against the SARS-CoV-2 internal nucleocapsid protein (anti-N IgG; Abcam, #ab274339, Cambridge, UK) and surface spike protein (anti-S IgG; Acro Biosystems, #RAS-T048, Newark, DE, US), were measured using commercially available ELISA kits according to the manufacturer’s instructions. The sVNT method was used to assess neutralizing antibodies against SARS-CoV-2 using the GenScript sVNT kit (GenScript; #L00847-A, Piscataway, NJ, USA). The sVNT detects neutralizing antibodies that block the interaction that mimics the binding between the viral spike protein and the host cell receptor via an ELISA-based method. The absorbance of a sample is inversely related to the titer of the anti-SARS-CoV-2 neutralizing antibodies, and the virus inhibition rate is calculated as follows: 100 * (1- (sample absorbance)/negative control absorbance)). The result was interpreted as positive for neutralizing antibodies if the percent value of sVNT inhibition met or exceeded 30% of the manufacturer’s cutoff. The percent value of sVNT inhibition was also analyzed. All experiments assessing anti-N IgG, anti-S IgG, and neutralizing antibodies were conducted twice in duplicate. If inconsistencies were found in antibody detection between the first and second experiments, a third experiment was performed. Among the two consistent experiments, the latter was selected for analysis.

### Measurement of cytokines

The cytokines known to be related to long COVID or pulmonary fibrosis, including interleukin-6 (IL-6; R&D systems, #D6050, Minneapolis, MN, USA), interleukin-10 (IL-10; R&D systems, #D1000B, Minneapolis, MN, USA), interleukin-1β (IL-1β; R&D systems, #DLB50, Minneapolis, MN, USA), and C-X-C Motif Chemokine Ligand 10/Interferon-gamma-inducible protein 10 (CXCL10/IP-10; R&D systems, #DIP100, Minneapolis, MN, USA), were evaluated using commercially available ELISA kits according to the manufacturer’s instructions.

### Statistical analysis

When comparing categorical variables, we used Pearson’s chi-square test if the number of cells with an expected frequency of less than five was less than 20% and Fisher’s exact test if it was greater than 20%. For continuous variables, the Mann‒Whitney U test was used if the assumption of normality was not satisfied by the Kolmogorov‒Smirnov test; if the assumption was met, Student’s t test was used. Variables with P < 0.2 in the univariable analyses and those with clinical importance were included in the backward stepwise multivariable logistic regression analysis, with 0.1 as the cutoff value for elimination to identify independent risk factors for long-term pulmonary sequelae. P < 0.05 was considered significant (SPSS, version 27.0; IBM Corp., Armonk, NY, USA).

### Ethics statement

This study was approved by the institutional review board of Boramae Medical Center (No. 26-2015-81 & 30-2023-61). Written informed consent was obtained from the study participants during both baseline enrollment and the 2^nd^ follow-up visit. Authors had access to information that could identify individual participants during the retrospective data collection. After data collection was completed, personal identifiers were removed prior before data processing. The study was conducted in accordance with the International Conference on Harmonization Guidelines for Good Clinical Practice and complied with the tenets of the Declaration of Helsinki.

## Results

### Baseline characteristics

A total of 107 patients were enrolled at baseline. These baseline patients were invited for a 2^nd^ follow-up visit, but 70 individuals were excluded for the following reasons: refusal to participate (n = 43), being unreachable (n = 26), or deceased (n = 1). Ultimately, 37 patients consented to the 2^nd^ follow-up visit (Fig 1). Among the 107 baseline patients, the median age was 51 (interquartile range [IQR] 43–61), and 57.9% were male (Table 1). For all patients, this was their first SARS-CoV-2 infection. The median duration from the onset of illness to admission was 5 days (IQR 3–7.5), and the median hospitalization period was 9 days (IQR 6.5–12.5), with a range of 3–41 days. The baseline serum sample for cytokines and antibody analysis was collected at a median of 24 days after symptom onset (IQR 20–30), with a range of 15–154 days. Overweight (34.6%, 37/107) and hypertension (33.6%, 36/107) were common underlying diseases, and 68.2% (73/107) of the patients were unvaccinated. During the hospitalization, 68 patients (63.6%) required oxygen via masks or nasal prongs, and two (1.9%) needed oxygen support via high-flow nasal cannulas. At the time of hospitalization, most patients (93.5%, 100/107) had pulmonary infiltration on chest X-ray, and 4.7% (5/107) had bacterial pneumonia. Among the patients, 66 (61.7%) received therapeutic remdesivir, 75 (70.1%) received steroid treatment, and 28 (26.2%) did not receive either therapy.

**Table 1:** Clinical characteristics of 107 patients hospitalized with COVID-19 at baseline.

Variables	Baseline patients (n = 107)
Age, years	51 (43-61)^*^
Male sex	62 (57.9)
Current smoker at admission	5 (4.7)
Days from onset of illness to diagnosis	2 (1-3)
Days from onset of illness to admission	5 (3-7.5)
Hospitalization period, days	9 (6.5-12.5)
Days from onset of illness to initial blood sampling	24 (20-30)
Underlying disease	
Overweight (30 > BMI ≥ 25)	37 (34.6)
Hypertension	36 (33.6)
Dyslipidemia	27 (25.2)
Diabetes mellitus	20 (18.7)
Obesity (BMI ≥ 30)	15 (14.0)
Psychiatric disorder	6 (5.6)
Cerebrovascular accident	2 (1.9)
Autoimmune disease	2 (1.9)
Bronchial asthma	1 (0.9)
Chronic kidney disease	1 (0.9)
Chronic liver disease	1 (0.9)
Hematologic malignancy	1 (0.9)
Solid organ transplantation	1 (0.9)
Under steroid	2 (2.7)
Under other immunosuppressant	3 (2.8)
SARS-CoV-2 vaccination status at hospitalization	
Unvaccinated	73 (68.2)
Partially vaccinated	23 (21.5)
Fully vaccinated	11 (10.3)
Maximum World Health Organization severity scale at hospitalization	
No oxygen requirement	37 (34.6)
Oxygen supply by mask or nasal prongs	68 (63.6)
Oxygen supply by high flow nasal cannula	2 (1.9)
Lung involvement on worst chest X-ray at hospitalization	
No pulmonary infiltration	7 (6.5)
With pulmonary infiltration	100 (93.5)
Days from onset of illness to worst chest X-ray	7 (6-9)
Combined bacterial pneumonia	5 (4.7)
Treatment at hospitalization	
Remdesivir (prophylactic)	8 (7.5)
Remdesivir (therapeutic)	66 (61.7)
Regdanvimab	7 (6.5)
Anticoagulant prophylaxis	57 (53.3)
Steroid	75 (70.1)
Duration, days	5 (4-9)
Maximum daily dose, mg^a^	6 (6-6)
Antibodies at baseline convalescence	
Positive for anti-N IgG	104 (97.2)
Positive for anti-S IgG	107 (100)
Positive for neutralizing antibodies	104 (97.2)
sVNT inhibition rate, %	96.7 (85.9-99.3)

BMI, body mass index; sVNT, surrogate virus neutralization test

* The result is the median value, the value in parentheses is the percentage, and the range is the interquartile range.

^a^Dose was converted to dexamethasone.

A total of 37 patients completed the 2^nd^ follow-up visit, with a median age of 54 years (IQR 47–62) (Table 2). The common underlying disease was dyslipidemia (43.2%, 16/37), followed by hypertension (40.5%, 15/37), overweight (35.1%, 13/37), and diabetes mellitus (27.0%, 10/37). At the time of hospitalization, 22 patients (59.5%) were unvaccinated, nine (24.3%) were partially vaccinated, and six (16.2%) were fully vaccinated. Oxygen therapy was required in 26 patients (70.3%), with 25 (67.6%) receiving it via masks or nasal prongs and one patient (2.7%) receiving it via a high-flow nasal cannula. At the time of hospitalization, bilateral pulmonary infiltrate was present in 89.2% (33/37) of patients, and it took a median of 7 days (IQR 5–8) from symptom onset to worst X-ray findings. At hospitalization, the median lymphocyte count was 839 cells/mm^3^ (IQR 708–1209), and the median C-reactive protein level was 5.51 mg/dL (IQR 2.60–9.96). During the hospitalization, therapeutic remdesivir was administered to 25 patients (67.6%), and 28 (75.7%) received steroids for a median of 7.5 days (IQR 4–9). Neither remdesivir nor steroids were administered to 8 patients (21.6%).

**Table 2:** Comparison of patients with a history of hospitalization due to COVID-19 depending on the presence of long-term pulmonary sequelae.

Variables	Second follow-up visit patients (n = 37)	Pulmonary sequelae (n = 22)	Without pulmonary sequelae (n = 15)	P
Age, years	54 (47-62)^*^	59 (51.5-66)	43 (38.5-53.5)	0.002
Male sex	20 (54.1)	14 (63.6)	6 (40.0)	0.157
Current smoker at hospitalization	1 (2.7)	–	1 (6.7)	0.405
Days from onset of illness to diagnosis	2 (1-4)	3 (1.75-4)	1 (1-4)	0.335
Days from onset of illness to admission	4 (3-6)	4.5 (3-6.75)	4 (2-5)	0.512
Hospitalization period, days	11 (8-15)	12 (9-16)	10 (7-12)	0.291
Days from onset of illness to convalescent blood sampling	24 (22-37)	26.5 (23-37.8)	23 (19.5-33)	0.133
Underlying disease				
Dyslipidemia	16 (43.2)	9 (40.9)	7 (46.7)	0.729
Hypertension	15 (40.5)	10 (45.5)	5 (33.3)	0.461
Overweight (30 > BMI ≥ 25)	13 (35.1)	7 (31.8)	6 (40.0)	0.609
Diabetes mellitus	10 (27.0)	6 (27.3)	4 (26.7)	>0.999
Obesity (BMI ≥ 30)	3 (8.1)	2 (9.1)	1 (6.7)	>0.999
Cerebrovascular accident	1 (2.7)	1 (4.5)	–	>0.999
Bronchial asthma	1 (2.7)	1 (4.5)	–	>0.999
Psychiatric disorder	5 (13.5)	3 (13.6)	2 (13.3)	>0.999
Chronic kidney disease	1 (2.7)	1 (4.5)	–	>0.999
SARS-CoV-2 vaccination status at hospitalization				
Unvaccinated	22 (59.5)	11 (50.0)	11 (73.3)	0.156
Partially vaccinated	9 (24.3)	6 (27.3)	3 (20.0)	0.711
Fully vaccinated	6 (16.2)	5 (22.7)	1 (6.7)	0.368
Maximum World Health Organization severity scale at hospitalization				
No oxygen requirement	11 (29.7)	7 (31.8)	4 (26.7)	>0.999
Oxygen supply by mask or nasal prongs	25 (67.6)	14 (63.6)	11 (73.3)	0.724
Oxygen supply by high flow nasal cannula	1 (2.7)	1 (4.5)	–	>0.999
Lung involvement on worst chest X-ray at hospitalization				
No pulmonary infiltration	2 (5.4)	1 (4.5)	1 (6.7)	>0.999
Unilateral pulmonary infiltration	2 (5.4)	1 (4.5)	1 (6.7)	>0.999
Bilateral pulmonary infiltration	33 (89.2)	20 (90.9)	13 (86.7)	>0.999
Days from onset of illness to worst chest X-ray	7 (5-8)	7 (5-8)	6.5 (5-8)	0.676
Laboratory findings at hospitalization^a^				
Lymphocyte count, cells/mm^3^	839 (708-1209)	825 (708-1082)	1084 (702-1409)	0.205
Aspartate aminotransferase, IU/L	39 (30-52)	41.5 (30-57.3)	34 (28.5-43)	0.412
Alanine aminotransferase, IU/L	42 (24-66)	45 (24.3-65.8)	35 (25-59.5)	0.938
C-reactive protein, mg/dL	5.51 (2.60-9.96)	6.03 (3.82-9.76)	2.63 (1.63-12.86)	0.279
Ferritin, *ng/mL*	309.5 (157.3-490.3)	290.5 (178-408.5)	309.5 (141-502.8)	>0.999
Lactate dehydrogenase, IU/L	292 (262-377)	275 (253-363)	307 (279.5-379)	0.430
Creatine kinase, U/L	84 (56.5-145.5)	72 (56.8-110.8)	84 (57-186)	0.317
D-dimer, mg/L	0.55 (0.39-0.83)	0.54 (0.39-0.76)	0.58 (0.41-0.86)	0.797
Fibrinogen, mg/dL	528.2 (402.1-633.2)	594.5 (400-672.9)	471.8 (458.5-540.4)	0.541
Treatment at hospitalization				
Remdesivir (prophylactic)	1 (2.7)	–	1 (6.7)	0.405
Remdesivir (therapeutic)	25 (67.6)	17 (77.3)	8 (53.3)	0.164
Regdanvimab	4 (10.8)	3 (13.6)	1 (6.7)	0.633
Anticoagulant prophylaxis	19 (51.4)	10 (45.5)	9 (60.0)	0.385
Steroid	28 (75.7)	17 (77.3)	11 (73.3)	>0.999
Duration, days	7.5 (4-9)	8 (4-13)	7 (5.5-9)	0.721
Maximum daily dose, mg^b^	6 (6-6)	6 (6-6)	6 (6-6)	0.630
Combined bacterial pneumonia	4 (10.8)	3 (13.6)	1 (6.7)	0.633

BMI, body mass index

* The result is the median value, the value in parentheses is the percentage, and the range is the interquartile range.

^a^Laboratory findings were evaluated among available data.

^b^Dose was converted to dexamethasone.

### Long-term pulmonary sequelae and baseline antibody status or cytokine levels

As observed on low-dose chest CT, long-term pulmonary sequelae were observed in 59.5% (22/37) of patients at the 2^nd^ visit. The median follow-up period between symptom onset during hospitalization and the follow-up visit was 27 months (IQR 25–28), with a range of 22–30 months (S1 Fig). Twenty patients (54.1%) were additionally diagnosed with COVID-19 without oxygen requirements at least once during the follow-up period (Table 3). The median time from the most recent infection to the 2^nd^ follow-up visit, considering the initial infections during hospitalization and any subsequent infections during the follow-up period was 20 months (IQR 10–26). At the 2^nd^ follow-up visit, one patient (2.7%) was unvaccinated, one (2.7%) was partially vaccinated, eight (21.6%) were fully vaccinated, and 27 (73.0) had received at least one booster dose.

**Table 3:** Radiologic and laboratory findings in patients with long-term pulmonary sequelae more than two years after hospitalization due to COVID-19.

Variables	Second follow-up visit patients (n = 37)	Pulmonary sequelae (n = 22)	Without pulmonary sequelae (n = 15)	P
Symptom onset at hospitalization to follow-up visit, months	27 (25-28)^*^	26.5 (25-28)	27 (25.5-27)	0.729
Current smoker at follow-up visit	5 (13.5)	4 (18.2)	1 (6.7)	0.629
Additional SARS-CoV-2 infection during follow-up period	20 (54.1)	13 (59.1)	7 (46.7)	0.457
Most recent infection to follow-up visit, months	20 (10-26)	16.5 (5.5-24.5)	22 (16-27)	0.162
Additional vaccination during follow-up period	33 (89.2)	19 (86.4)	14 (93.3)	0.633
SARS-CoV-2 vaccination status at follow-up visit				
Unvaccinated	1 (2.7)	1 (4.5)	–	>0.999
Partially vaccinated	1 (2.7)	1 (4.5)	–	>0.999
Fully vaccinated	8 (21.6)	4 (18.2)	4 (26.7)	0.690
At least one booster dose	27 (73.0)	16 (72.7)	11 (73.3)	>0.999
Resting percutaneous oxygen saturation, %	98 (97-98)	98 (97-98)	97 (97-98.5)	0.809
Respiratory symptom at second follow-up visit				
Dyspnea	9 (24.3)	6 (27.3)	3 (20.0)	0.711
mMRC grade 1	7 (77.8)	4 (66.7)	3 (100)	0.500
mMRC grade 2	2 (22.2)	2 (33.3)	–	0.500
Dyspnea 12 scale	4 (2-8)	4.5 (2.25-13.5)	4 (3-6)	0.485
Cough	5 (13.5)	2 (9.1)	3 (20.0)	0.377
Sputum	4 (10.8)	1 (4.5)	3 (20.0)	0.283
Low dose chest CT findings at second follow-up visit				
Fibrotic or fibrotic-like changes	15 (40.5)	15 (68.2)	–	–
Traction bronchiectasis	3 (8.1)	3 (13.6)	–	–
Honeycombing	–	–	–	–
Parenchymal band	8 (21.6)	8 (36.4)	–	–
Linear atelectasis	6 (16.2)	6 (27.3)	–	–
Bronchiectasis	4 (10.8)	4 (18.2)	–	–
Ground glass opacity	12 (32.4)	12 (54.5)	–	–
Unilateral	2 (16.7)	2 (16.7)	–	–
Bilateral	10 (83.3)	10 (83.3)	–	–
Number of involved lobes	5 (3-5)	5 (3-5)	–	–
Cytokines at baseline convalescence				
Detection for interleukin 6	6 (16.2)	3 (13.6)	3 (20.0)	0.670
Interleukin 6, pg/mL	0 (0−0)	0 (0−0)	0 (0−0)	0.772
Detection for interleukin 10	31 (83.8)	18 (81.8)	13 (86.7)	>0.999
Interleukin 10, pg/mL	3.47 (1.99-5.01)	3.13 (1.89-3.96)	4.13 (2.35-6.94)	0.154
Detection for interleukin 1beta	–	–	–	–
Detection for CXCL10/IP-10	37 (100)	22 (100)	15 (100)	–
CXCL10/IP-10, pg/mL	101.91 (87.89-147.94)	106.83 (88.27-153.69)	101.91 (82.07-130.38)	0.734
Antibodies at baseline convalescence				
Positive for anti-N IgG	36 (97.3)	21 (95.5)	15 (100)	>0.999
Positive for anti-S IgG	37 (100)	22 (100)	15 (100)	–
Positive for neutralizing antibodies	35 (94.6)	22 (100)	13 (86.7)	0.158
sVNT inhibition rate, %	96.6 (79.9-99.3)	98.2 (91.5-99.3)	86.5 (58.7-98.7)	0.041
Antibodies at second follow-up visit				
Positive for anti-N IgG	37 (100)	22 (100)	15 (100)	–
Positive for anti-S IgG	37 (100)	22 (100)	15 (100)	–
Positive for neutralizing antibodies	37 (100)	22 (100)	15 (100)	–
sVNT inhibition rate, %	99.2 (98.4-99.2)	99.1 (98.4-99.2)	99.1 (98.5-99.3)	0.621

mMRC, modified medical research council dyspnea scale; CT, computed tomography; CXCL10/IP-10, C-X-C motif chemokine ligand 10/interferon-gamma-inducible protein 10; sVNT, surrogate virus neutralization test

* The result is the median value, the value in parentheses is the percentage, and the range is the interquartile range.

The median resting SpO_2_ in all patients was 98% (IQR 97–98), and nine (24.3%) reported persistent dyspnea. Among these, seven patients were classified as mMRC grade 1 and two as mMRC grade 2. Only 27.3% (6/22) of patients with pulmonary sequelae reported dyspnea, and there was no significant difference in the prevalence of dyspnea between the two groups. Fifteen patients (40.5%) had fibrotic or fibrotic-like pulmonary changes, which included traction bronchiectasis (8.1%, 3/37), parenchymal bands (21.6%, 8/37), linear atelectasis (16.2%, 6/37), and bronchiectasis (10.8%, 4/37). GGO findings on chest CT were observed in 32.4% (12/37) of patients, with bilateral distribution in 83.3% (10/12) and involvement of a median of 5 lobes (IQR 3–5). In additional analysis based on follow-up period after hospitalization, fibrotic or fibrotic-like changes were more common in patients with follow up periods of < 2 years compared to those with ≥ 2 years (83.3%, 5/6 vs. 32.3%, 10/31; P = 0.031, S2 Table).

Baseline convalescent serum was obtained at a median of 24 days (IQR 22–37) after symptom onset, with a range of 18–154 days (Table 2). Anti-N IgG and anti-S IgG were present in 97.3% (36/37) and 100% (37/37) of the baseline serum samples, respectively (Table 3). The positivity for neutralizing antibodies was not significantly different between the two groups when the cutoff value of 30% was applied (100%, 22/22 vs. 86.7%, 13/15; P = 0.158). The percent value of sVNT inhibition was higher in the pulmonary sequelae group than in the nonpulmonary sequelae group (median 98.2% [IQR 91.5–99.3] vs. 86.5% [IQR 58.7–98.7], P = 0.041). At the 2^nd^ visit, all patients were positive for anti-N IgG, anti-S IgG, and neutralizing antibodies, with no difference in the percent value of sVNT inhibition between the two groups (median 99.1% [IQR 98.4–99.2] vs. 99.1% [IQR 98.5–99.3], P = 0.621).

**Fig 2:**
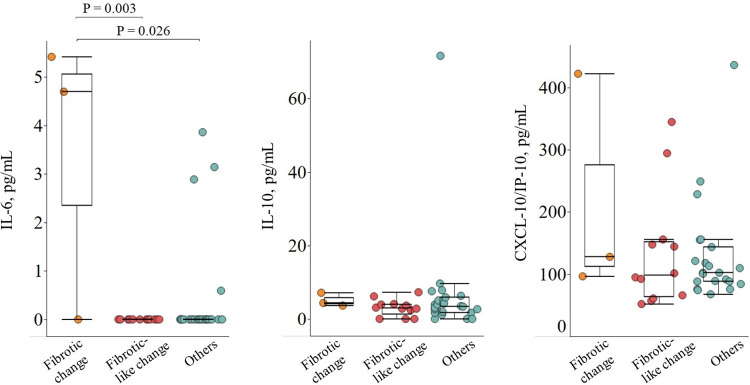
Convalescent cytokines levels in patients hospitalized with SARS-CoV-2 infection according to pulmonary fibrotic sequelae. Cytokine levels are shown as box plots with interquartile ranges. P values less than 0.05 are presented at the top, indicating a comparison between the groups. IL-6, interleukin-6; IL-10, interleukin-10; CXCL-10/IP-10, C-X-C motif chemokine ligand 10/interferon-gamma-inducible protein 10.

The baseline convalescent serum levels of cytokines, including IL-6, IL-10, and CXCL-10/IP-10, did not differ between patients with pulmonary sequelae and those without (Table 3). IL-1β was not detected at baseline convalescent serum in any of the 2^nd^ visit patients. An additional comparison was conducted based on detailed radiologic findings focused on fibrotic sequelae to investigate the associations between cytokines involved in IPF and post-COVID-19 pulmonary fibrotic sequelae. Patients at the 2^nd^ visit were categorized into three groups: fibrotic change (n = 3), fibrotic-like change (n = 12), and others (n = 22), and these groups were compared. The ‘others’ group included 7 patients with GGOs and 15 without pulmonary sequelae. IL-6 levels were higher in patients with traction bronchiectasis (n = 3) in the fibrotic change group (median 4.698 pg/mL [IQR 2.349–5.056]) than in those in the fibrotic-like change group (median 0 pg/mL [IQR 0–0], P = 0.003) or others (median 0 pg/mL [IQR 0–0], P = 0.026; Fig 2). There were no significant differences in IL-10 or CXCL-10/IP-10 levels among the three groups.

### Factors associated with long-term pulmonary sequelae

The pulmonary sequelae group was older than the non-pulmonary sequelae group (59 years [IQR 51.5–66] vs. 43 years [IQR 38.5–53.5]; P = 0.002, Table 2). The groups were similar in the other baseline characteristics, including underlying diseases, vaccination status, maximum WHO severity scale score, radiologic and laboratory findings, and treatment (Table 2). Multivariable analysis was performed to identify factors associated with long-term pulmonary sequelae (Table 4). Pulmonary sequelae were found to be associated only with older age (adjusted odds ratio 1.130, 95% confidence interval 1.028–1.243; P = 0.011).

**Table 4:** Factors associated with long-term pulmonary sequelae in patients with a history of hospitalization due to COVID-19 according to multivariable analysis.

Variables	Adjusted odds ratio (95% Confidence interval)	P
Age, year	1.130 (1.028-1.243)	0.011

### Long COVID-related symptoms

At the 2^nd^ visit, long COVID-related symptoms and the degree of physical and mental recovery were investigated (S1 Table). There were 24 patients (64.9%) who reported long COVID-related symptoms, with the median number of involved systems per patient being 2.5 (IQR 1–4) among the five systems: general, respiratory, cardiac, gastrointestinal, and psycho-neurological symptoms. The most common symptom was fatigue (40.5%, 15/37), followed by dyspnea (24.3%, 9/27) and problems with memory (24.3%, 9/27). Among the symptoms of the five systems, respiratory symptoms (48.6%, 18/37), such as dyspnea, hoarseness, cough, sputum, and rhinorrhea, were the most frequent. In the relationship analysis between long COVID and positivity for neutralizing antibodies, no significant difference was identified (patients with at least one long COVID-related symptom vs. patients without any symptoms; 91.7% (22/24) vs. 100% (13/13), P = 0.532).

## Discussion

This study evaluated the immune status and cytokine responses of patients hospitalized with SARS-CoV-2 infection during the convalescent period and their associations with long-term pulmonary sequelae more than two years later. Long-term pulmonary sequelae, which included fibrotic, fibrotic-like, and GGO findings, were present in 59.5% (22/37) of the study patients. Older age was associated with long-term pulmonary sequelae.

Multivariable analysis revealed that long-term pulmonary sequelae were associated with older age, which is a well-established risk factor for severe COVID-19 and IPF. Previous studies identified it as a risk factor for residual lung abnormalities up to 12 months after SARS-CoV-2 infection [8,18]. According to the univariable analysis, the percent value of sVNT inhibition was higher in patients with pulmonary sequelae. However, the manufacturer recommended qualitatively interpreting the percent value of sVNT inhibition with a cutoff of 30%. Although the quantitatively interpreted 50% inhibition titer of sVNT was moderately correlated with conventional VNT_50_ and pseudovirus-based VNT_50_ titers, with R^2^ values ranging from 0.6548 to 0.8591 and 0.4937 to 0.8374, respectively [7,19], compared with conventional VNT, sVNT assays presented a relatively narrow detection range and early saturation at high titers [20]. Further studies are needed to better elucidate the relationship between the percent value of sVNT inhibition and long-term pulmonary sequelae using quantitative sVNT kits.

The sVNT method has clear, practical advantages for clinical settings and provides standardized and comparable results across different laboratories. However, the sVNT method has several limitations. First, the sVNT method only assesses neutralizing antibodies that inhibit the interaction between the spike protein’s receptor-binding domain (RBD) and the angiotensin-converting enzyme 2 receptor on host cells. It cannot detect neutralizing antibodies targeting non-RBD regions, such as the N-terminal domain or S2 proteins. However, since most neutralizing antibodies target the RBD, the sVNT method likely captures clinically meaningful neutralizing antibodies [21]. Second, we used the wild-type horseradish peroxidase-conjugated recombinant SARS-CoV-2 RBD fragment provided with the commercial kit. Patients included in this study were presumed to have been infected between February 2021 and April 2022 based on the date of symptom onset: 19 during the wild-type epidemic, 85 during the Delta variant epidemic, and 3 during the Omicron variant epidemic [22]. When neutralizing antibodies are evaluated via the sVNT method, the measured neutralizing antibody level against the Omicron variant is reported to be lower than that against the wild type; however, no significant difference was observed between the Delta variant and the wild type [23]. Therefore, the impact of confounding due to variant virus infection is likely minimal.

In the analysis of cytokines related to the inflammatory response, the comparison between groups using more detailed radiologic findings focused on pulmonary fibrosis (fibrotic findings vs. fibrotic-like findings vs. others) revealed significantly higher levels of convalescent IL-6 in patients with fibrotic findings than in those with fibrotic-like findings and others (Fig 2). This was consistent with previous reports that higher IL-6 was associated with post-COVID-19 pulmonary fibrosis and IPF [9,24].

The frequency of pulmonary sequelae after SARS-CoV-2 infection varies widely, depending on the timing of the studies and the disease severity of the included subjects. In studies conducted before the introduction of vaccination and specific treatments for COVID-19, chest CT after two year of infection revealed abnormal findings in 38.9% (56/144) and 82.5% (47/57) of patients, which included 13.2% requiring noninvasive mechanical ventilation and 47.4% requiring noninvasive or invasive mechanical ventilation, respectively [2,3]. There are limited data on long-term pulmonary sequelae following the introduction of vaccination and antiviral treatment. In one study, which included 32% (16/50) of patients treated with remdesivir and, 64.0% (32/50) of patients requiring noninvasive mechanical ventilation, 8.2% (4/49) had fibrotic lesions, and 38.8% (19/49) presented GGO findings on chest CT evaluated two years after infection [25]. In another study, which included 28.6% (24/84) of patients treated with antiviral agents and 53.6% (45/84) of patients requiring oxygen supply beyond that provided via nasal cannulas or masks, 7.1% (6/84) had fibrotic abnormalities, and 2.4% (2/84) had GGOs on chest CT after one year of COVID-19 [10].

The prevalence of abnormal findings on chest CT in this study was relatively high, with 8.1% (3/37) showing fibrotic abnormalities and 32.4% (12/37) exhibiting GGO findings. These figures may have been overestimated due to selection bias, as patients who consented to the 2^nd^ visit for further evaluation may have had more severe cases. Compared with non-returning patients, 2^nd^ visit patients had a more extended hospitalization period and a greater prevalence of dyslipidemia and psychiatric disorders (S3 Table). The duration of steroid administration was longer, and bacterial pneumonia was more common in 2^nd^ visit patients. However, the two groups had similar vaccination status, maximum WHO severity scale, radiological findings, and laboratory findings. In this study, most patients (97.3%, 36/37) did not require an oxygen supply beyond that provided via nasal cannulas or masks, indicating a lower severity of COVID-19 than that reported in previous studies. This might be due to the high percentage of patients receiving antiviral treatment (67.6%, 25/37), which could have prevented further exacerbations requiring higher oxygen levels. In the additional comparison based on follow-up period after hospitalization, a higher percentage of patients with follow up periods of < 2 years had fibrotic or fibrotic-like pulmonary sequelae compared to those with ≥ 2 years (S2 Table). These findings are consistent with previous observations indicating a gradual resolution of pulmonary sequelae over time [2,25].

Among patients with pulmonary sequelae, SpO2 ranged from 95% to 99%, and most (72.7%, 16/22) did not complain of dyspnea (Table 3). Although pulmonary sequelae have been linked to respiratory symptoms and functional abnormalities, such as a reduced diffusing capacity for carbon monoxide [2], this study did not find a significant difference in respiratory discomfort between those with and without pulmonary sequelae. The clinical significance of asymptomatic pulmonary sequelae remains unclear, and our results support the guidance of the British Thoracic Society and WHO that routine chest CT follow-up is not recommended in asymptomatic patients who have recovered from mild COVID-19 [26,27].

In previous studies involving subjects aged 55 years and older who were current or former smokers undergoing low-dose chest CT screening, GGOs, and fibrotic lesions were identified in 21.3% of cases, and reticulation and fibrotic lesions were reported in 20.1% [28,29]. This study identified pulmonary sequelae, including GGOs and fibrotic changes, in 59.5% of patients (22/37) even two years after COVID-19-related hospitalization. Among them, only 27.3% of patients (6/22) reported experiencing persistent dyspnea. In a context where medical checkups are frequently conducted, confirming a history of past COVID-19 infection will assist in interpreting incidental findings observed by imaging.

On the 2^nd^ visit, patients were interviewed in detail about long COVID-related symptoms and their impact on daily life. Twenty-four patients (64.9%) complained of long COVID-related symptoms after two years of infection, similar to the 59.7% (215/360) reported previously [30]. The most common symptom was fatigue, followed by memory issues, consistent with a previous report [30].

The strength of this study is that it presented valuable data on long-term pulmonary sequelae during the period following the introduction of vaccination and targeted treatments for COVID-19. Patients with mild to moderate COVID-19 treated with remdesivir exhibited a high prevalence of long-term pulmonary sequelae. In this study, the associations between convalescent neutralizing antibodies or cytokine levels and long-term pulmonary sequelae could not be established. However, further research is needed to assess the utility of the quantitatively interpreted percent value of sVNT inhibition in predicting long-term pulmonary sequelae.

This study has several limitations. First, the small sample size may affect the statistical power of the analysis. Second, due to the study design requiring a hospital visit, patients with more severe COVID-19 at hospitalization might selectively participate in this study. We compared 2^nd^ visit patients and nonreturning patients (S3 Table). Third, chest CT scans were not performed during the SARS-CoV-2-related hospitalization. Additionally, 54.1% of the 2^nd^ visit patients (20/37) experienced additional SARS-CoV-2 infections during the follow-up period (Table 3), making it challenging to determine whether the abnormal findings on chest CT identified in this study were related to previous SARS-CoV-2-related hospitalizations. However, only one person with pulmonary sequelae had underlying respiratory disease (Table 2). Additionally, there were no significant differences in the presence of additional SARS-CoV-2 infections or the time from the most recent infection to the 2^nd^ follow-up visit between the groups with and without pulmonary sequelae (Table 3).

Furthermore, no SARS-CoV-2 reinfections during the follow-up period required oxygen therapy, suggesting that any additional infections had minimal impact on radiologic abnormalities. Fourth, since quantitative analyses of chest CT and pulmonary function tests were not performed, there were insufficient aspects to evaluate the severity of pulmonary sequelae. We attempted to address this shortage by evaluating complaints of dyspnea using the mMRC grade and Dyspnea 12 scale. Fifth, variant information for the infected patients was not identified.

## Conclusion

Pulmonary sequelae, including fibrotic, fibrotic-like, and GGO findings on CT scans after two years of mild to moderate COVID-19, were common (59.5%, 22/37). However, neutralizing antibody assays using sVNT and cytokine levels during recovery did not predict these sequelae. Instead, older age was significantly associated with these variables. Two-year pulmonary sequelae in patients with mild to moderate COVID-19 during the vaccination and active treatment eras are primarily asymptomatic and may not warrant further medical intervention.

## Supporting information

S1 Table Long COVID more than two years after hospitalization due to COVID-19. (DOCX)

S2 Table Comparison of long-term pulmonary sequelae due to COVID-19 between patients with follow up periods of < 2 years and ≥ 2 years. (DOCX)

S3 Table Comparison of clinical characteristics of hospitalization due to COVID-19 between second visit patients and non-returning patients. (DOCX)

S1 Fig Number of patients by duration from symptom onset at hospitalization to 2^nd^ follow-up visit. Each circle represents one patient.
(TIF)
