# Supplementary material for: Long-term pulmonary sequelae and convalescent immune reactions in mild to moderate COVID-19 patients during the active treatment era
Source: PLoS One. 2025 Jun 5;20(6):e0325379. doi: 10.1371/journal.pone.0325379 (PMC12140412; doi:10.1371/journal.pone.0325379)
Supplement: S1 Table — (DOCX) [file pone.0325379.s001.docx]

S1 Table. Long COVID more than two years after hospitalization due to COVID-19

| Variables | All patients  (n=37) |
| --- | --- |
| Weight gain | 34 (91.9) |
| Increase in BMI, kg/m^2^ | 1.901 (1.273-2.507) |
| Overweight (BMI≥25) | 16 (43.3) |
| Obesity (BMI≥30) | 6 (16.2) |
| Changes in occupation or working hours | 2 (5.4) |
| Physical performance had not fully recovered to pre-hospitalization | 23 (62.2) |
| Recovered physical performance compared to before hospitalization, % | 80 (70-90) |
| Mental performance had not fully recovered to pre-hospitalization | 16 (43.2) |
| Recovered mental performance compared to before hospitalization, % | 80 (70-90) |
| Presence of symptoms related to long COVID | 24 (64.9) |
| The number of involved systems | 2.5 (1-4) |
| General symptoms | 15 (40.5) |
| Fatigue | 15 (40.5) |
| FACIT-fatigue scale | 20 (12.5-26) |
| Myalgia | 7 (18.9) |
| Faces Pain Scale | 5 (3.5-5.5) |
| Febrile sense | - |
| Respiratory symptoms | 18 (48.6) |
| Dyspnea | 9 (24.3) |
| mMRC grade 1 | 7 |
| mMRC grade 2 | 2 |
| Dyspnea 12 scale | 4 (2-8) |
| Hoarseness | 5 (13.5) |
| Cough | 5 (13.5) |
| Sputum | 4 (10.8) |
| Rhinorrhea | 2 (5.4) |
| Cardiovascular symptoms | 8 (21.6) |
| Chest pain | 5 (13.5) |
| Chest tightness | 5 (13.5) |
| Palpation | 3 (8.1) |
| Gastrointestinal symptoms | 8 (21.6) |
| Dyspepsia | 6 (16.2) |
| Nausea | 2 (5.4) |
| Anorexia | 1 (2.7) |
| Abdominal pain | - |
| Changes in bowel habits | - |
| Psycho-neurological symptoms | 15 (40.5) |
| Problem with Memory | 9 (24.3) |
| Problem with Concentration | 7 (18.9) |
| Olfactory disorder | 7 (18.9) |
| Taste disorder | 2 (5.4) |
| Depression | 5 (13.5) |
| Anxiety | 3 (8.1) |
| Generalized Anxiety Disorder-7 score | 7 (6.5-13) |
| Dizziness | 4 (10.8) |
| Headache | 2 (5.4) |

BMI, body mass index; FACIT-fatigue scale, Functional Assessment of Chronic Illness Therapy-Fatigue scale; mMRC; modified Medical Research Council dyspnea scale

^*^The result is the median value, the value in parentheses is the percentage, and the range is the interquartile range.
