# Supplementary material for: Long-term pulmonary sequelae and convalescent immune reactions in mild to moderate COVID-19 patients during the active treatment era
Source: PLoS One. 2025 Jun 5;20(6):e0325379. doi: 10.1371/journal.pone.0325379 (PMC12140412; doi:10.1371/journal.pone.0325379)
Supplement: S2 Table — (DOCX) [file pone.0325379.s002.docx]

S2 Table. Comparison of long-term pulmonary sequelae due to COVID-19 between patients with follow up periods of < 2 years and ≥ 2 years.

| Variables | Second follow-up visit patients  (n=37) | Follow-up period < 2 years  (n=6) | Follow-up period ≥ 2 years (n=31) | P |
| --- | --- | --- | --- | --- |
| Current smoker at follow-up visit | 5 (13.5) | 1 (16.7) | 4 (12.9) | >0.999 |
| Additional SARS-CoV-2 infection during follow-up period | 20 (54.1) | 2 (33.3) | 18 (58.1) | 0.383 |
| Most recent infection to follow-up visit, months | 20 (10-26) ^*^ | 22.5 (19-23) | 19 (7.5-27) | 0.967 |
| Resting percutaneous oxygen saturation, % | 98 (97-98) | 99 (97-98) | 98 (97-98) | 0.415 |
| Respiratory symptom at second follow-up visit | | | | |
| Dyspnea | 9 (24.3) | 2 (33.3) | 7 (22.6) | 0.620 |
| mMRC grade 1 | 7 (77.8) | 1 (50.0) | 6 (85.7) | 0.417 |
| mMRC grade 2 | 2 (22.2) | 2 (50.0) | 1 (14.3) | 0.417 |
| Dyspnea 12 scale | 4 (2-8) | 12 (7-17) | 3 (2-6) | 0.475 |
| Cough | 5 (13.5) | - | 5 (16.1) | 0.567 |
| Sputum | 4 (10.8) | - | 4 (12.9) | >0.999 |
| Low dose chest CT findings at second follow-up visit | | | | |
| Pulmonary sequelae | 22 (59.5) | 4 (83.3) | 17 (54.8) | 0.368 |
| Fibrotic or fibrotic-like changes | 15 (40.5) | 5 (83.3) | 10 (32.3) | 0.031 |
| Traction bronchiectasis | 3 (8.1) | 2 (33.3) | 1 (3.2) | 0.062 |
| Honeycombing | - | - | - | - |
| Parenchymal band | 8 (21.6) | 1 (16.7) | 7 (22.6) | >0.999 |
| Linear atelectasis | 6 (16.2) | 3 (50.0) | 3 (9.7) | 0.042 |
| Bronchiectasis | 4 (10.8) | 1 (16.7) | 3 (9.7) | 0.524 |
| Ground glass opacity | 12 (32.4) | 2 (33.3) | 10 (32.3) | >0.999 |
| Unilateral | 2 (16.7) | - | 2 (20.0) | >0.999 |
| Bilateral | 10 (83.3) | 2 (100.0) | 8 (80.0) | >0.999 |
| Number of involved lobes | 5 (3-5) | 5 (4-5) | 4 (2-5) | 0.565 |

mMRC, modified medical research council dyspnea scale; CT, computed tomography

^*^The result is the median value, the value in parentheses is the percentage, and the range is the interquartile range.
