# Supplementary material for: Long-term pulmonary sequelae and convalescent immune reactions in mild to moderate COVID-19 patients during the active treatment era
Source: PLoS One. 2025 Jun 5;20(6):e0325379. doi: 10.1371/journal.pone.0325379 (PMC12140412; doi:10.1371/journal.pone.0325379)
Supplement: S3 Table — (DOCX) [file pone.0325379.s003.docx]

S3 Table. Comparison of clinical characteristics of hospitalization due to COVID-19 between second visit patients and non-returning patients

| Variables | All patients  (n=107) | Second visit patients  (n=37) | Non-returning patients (n=70) | P |
| --- | --- | --- | --- | --- |
| Age, years | 51 (43-61)^*^ | 54 (47-62) | 49.5 (40.3-59) | 0.077 |
| Male sex | 62 (57.9) | 20 (54.1) | 42 (60.0) | 0.553 |
| Current smoker at hospitalization | 5 (4.7) | 1 (2.7) | 4 (5.7) | 0.657 |
| Days from onset of illness to diagnosis | 2 (1-3) | 2 (1-4) | 2 (1-3) | 0.514 |
| Days from onset of illness to admission | 5 (3-7.5) | 4 (3-6) | 6 (3-8) | 0.039 |
| Hospitalization period, days | 9 (6.5-12.5) | 11 (8-15) | 8 (6-11.8) | 0.003 |
| Underlying disease |  |  |  |  |
| Overweight (30>BMI≥25) | 37 (34.6) | 13 (35.1) | 24 (34.3) | 0.930 |
| Hypertension | 36 (33.6) | 15 (40.5) | 21 (30.0) | 0.272 |
| Dyslipidemia | 27 (25.2) | 16 (43.2) | 11 (15.7) | 0.002 |
| Diabetes mellitus | 20 (18.7) | 10 (27.0) | 10 (14.3) | 0.108 |
| Obesity (BMI≥30) | 15 (14.0) | 3 (8.1) | 12 (17.1) | 0.200 |
| Psychiatric disorder | 6 (5.6) | 5 (13.5) | 1 (1.4) | 0.018 |
| Cerebrovascular accident | 2 (1.9) | 1 (2.7) | 1 (1.4) | >0.999 |
| Bronchial asthma | 1 (0.9) | 1 (2.7) | - | 0.346 |
| Autoimmune disease | 2 (1.9) | - | 2 (2.9) | 0.543 |
| Chronic kidney disease | 1 (0.9) | 1 (2.7) | - | 0.346 |
| Chronic liver disease | 1 (0.9) | - | 1 (1.4) | >0.999 |
| Hematologic malignancy | 1 (0.9) | - | 1 (1.4) | >0.999 |
| Solid organ transplantation | 1 (0.9) | - | 1 (1.4) | >0.999 |
| Under steroid | 2 (2.7) | - | 2 (2.9) | 0.543 |
| Under other immunosuppressant | 3 (2.8) | - | 3 (4.3) | 0.550 |
| SARS-Cov-2 vaccination status at hospitalization | | | | |
| Unvaccinated | 73 (68.2) | 22 (59.5) | 51 (72.9) | 0.157 |
| Partially vaccinated | 23 (21.5) | 9 (24.3) | 14 (20.0) | 0.605 |
| Fully vaccinated | 11 (10.3) | 6 (16.2) | 5 (7.1) | 0.184 |
| Maximum World Health Organization severity scale at hospitalization | | | | |
| No oxygen requirement | 37 (34.6) | 11 (29.7) | 26 (37.1) | 0.443 |
| Oxygen supply by mask or nasal prongs | 68 (63.6) | 25 (67.6) | 43 (61.4) | 0.530 |
| Oxygen supply by high flow nasal cannula | 2 (1.9) | 1 (2.7) | 1 (1.4) | >0.999 |
| Lung involvement on worst chest x-ray at hospitalization | | | | |
| No pulmonary infiltration | 7 (6.5) | 2 (5.4) | 5 (7.1) | >0.999 |
| With pulmonary infiltration | 100 (93.5) | 35 (94.6) | 65 (92.9) | >0.999 |
| Days from onset of illness to worst chest x-ray | 7 (6-9) | 7 (5-8) | 7 (6-9) |  |
| Laboratory findings at hospitalization |  |  |  |  |
| Lymphocyte count, cells/mm^3^ | 936 (708-1194) | 839 (708-1209) | 954 (731-1175) | 0.556 |
| Aspartate aminotransferase, IU/L | 40 (40-70.5) | 39 (30-52) | 41 (33-73) | 0.253 |
| Alanine aminotransferase, IU/L | 42 (24.5-71.5) | 42 (24-66) | 42.5 (26.5-78.8) | 0.527 |
| C-reactive protein, mg/dL | 5.99 (3.395-9.655) | 5.51 (2.60-9.96) | 6.15 (3.90-8.97) | 0.621 |
| Ferritin*,* ng/mL | 249 (207.3-890.8) | 309.5 (157.3-490.3) | 378 (215-991.3) | 0.157 |
| Lactate dehydrogenase, IU/L | 317 (267-392.5) | 292 (262-377) | 328 (268-406) | 0.271 |
| Creatine kinase, U/L | 84 (58-148) | 84 (56.5-145.5) | 84.5 (59.3-148) | 0.566 |
| D-dimer, mg/L | 0.54 (0.37-0.77) | 0.55 (0.39-0.83) | 0.54 (0.35-0.76) | 0.327 |
| Fibrinogen, mg/dL | 516 (446-657.7) | 528.2 (402.1-633.2) | 514 (460.1-657.7) | 0.760 |
| Treatment at hospitalization |  |  |  |  |
| Remdesivir (prophylactic) | 8 (7.5) | 1 (2.7) | 7 (10.0) | 0.258 |
| Remdesivir (therapeutic) | 66 (61.7) | 25 (67.6) | 41 (58.6) | 0.363 |
| Regdanvimab | 7 (6.5) | 4 (10.8) | 3 (4.3) | 0.232 |
| Anticoagulant prophylaxis | 57 (53.3) | 19 (51.4) | 38 (54.3) | 0.772 |
| Steroid | 75 (70.1) | 28 (75.7) | 47 (67.1) | 0.359 |
| Duration, days | 5 (4-9) | 7.5 (4-9) | 5 (4-7.5) | 0.032 |
| Maximum daily dose, mg^b^ | 6 (6-6) | 6 (6-6) | 6 (6-6) | 0.517 |
| Combined bacterial pneumonia | 5 (4.7) | 4 (10.8) | 1 (1.4) | 0.048 |
| Days from onset of illness to convalescent blood sampling | 24 (20-30) | 24 (22-37) | 22.5 (20-28) | 0.133 |
| Antibodies at baseline convalescence |  |  |  |  |
| Positive for anti-N IgG | 104 (97.2) | 36 (97.3) | 68 (97.1) | >0.999 |
| Positive for anti-S IgG | 107 (100) | 37 (100) | 70 (100) | - |
| Positive for neutralizing antibodies | 104 (97.2) | 35 (94.6) | 69 (98.6) | 0.274 |
| sVNT inhibition rate, % | 96.4 (83.6-99.2) | 96.6 (79.9-99.3) | 96.3 (86.1-99.2) | 0.839 |

BMI, body mass index; sVNT, surrogate virus neutralization test

^*^The result is the median value, the value in parentheses is the percentage, and the range is the interquartile range

^a^ Laboratory findings were evaluated among available data.

^b^ The dose converted to dexamethasone.
