# Supplementary figures and images for: Long-term pulmonary sequelae and convalescent immune reactions in mild to moderate COVID-19 patients during the active treatment era
Source: PLoS One. 2025 Jun 5;20(6):e0325379. doi: 10.1371/journal.pone.0325379 (PMC12140412; doi:10.1371/journal.pone.0325379)

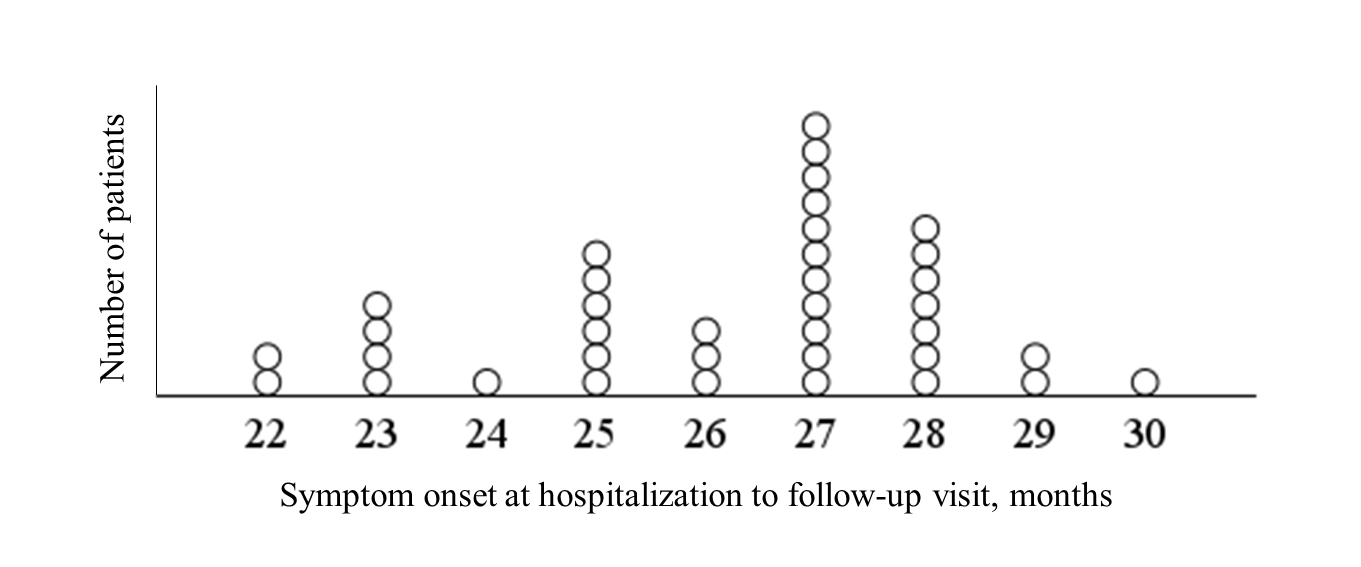

Supplement: S1 Fig — Each circle represents one patient. (TIF) [file pone.0325379.s004.tif]
